# The Influence of Manganese and Glutamine Intake on Antioxidants and Neurotransmitter Amino Acids Levels in Rats’ Brain

**DOI:** 10.1007/s11064-016-1928-7

**Published:** 2016-05-09

**Authors:** Maria Szpetnar, Dorota Luchowska-Kocot, Anna Boguszewska-Czubara, Jacek Kurzepa

**Affiliations:** Chair and Department of Medical Chemistry, Medical University of Lublin, 4A Chodźki Street, 20-093 Lublin, Poland

**Keywords:** Manganese, Glutamine, Antioxidants, Oxidative stress, Neurotransmitter amino acids

## Abstract

Depending on the concentration, Mn can exert protective or toxic effect. Potential mechanism for manganese neurotoxicity is manganese-induced oxidative stress. Glutamine supplementation could reduce manganese-induced neurotoxicity and is able to influence the neurotransmission processes. The aim of this study was to investigate whether the long term administration of manganese (alone or in combination with glutamine) in dose and time dependent manner could affect the selected parameters of oxidative-antioxidative status (superoxide dismutase and glutathione peroxidase activities, concentrations of vitamin C and malonic dialdehyde) and concentrations of excitatory (Asp, Glu) and inhibitory amino acids (GABA, Gly) in the brain of rats. The experiments were carried out on 2-months-old albino male rats randomly divided into 6 group: Mn300 and Mn500—received solution of MnCl_2_ to drink (dose 300 and 500 mg/L, respectively**)**, Gln group—solution of glutamine (4 g/L), Mn300-Gln and Mn500-Gln groups—solution of Mn at 300 and 500 mg/L and Gln at 4 g/L dose. The control group (C) received deionized water. Half of the animals were euthanized after three and the other half—after 6 weeks of experiment. The exposure of rats to Mn in drinking water contributes to diminishing of the antioxidant enzymes activity and the increase in level of lipid peroxidation. Glutamine in the diet admittedly increases SOD and GPx activity, but it is unable to restore the intracellular redox balance. The most significant differences in the examined amino acids levels in comparison to both control and Gln group were observed in the group of rats receiving Mn at 500 mg/L dose alone or with Gln. It seems that Gln is amino acid which could improve antioxidant status and affect the concentrations of the neurotransmitters.

## Introduction

Manganese (Mn) is an essential trace element for humans, animals and plants. It is required for normal growth, development and cellular homeostasis [1]. Mn participates in numerous enzymatic reactions, including the synthesis of amino acids, proteins, lipids and carbohydrates [2]. Additionally, this element is necessary for normal immune function, adenosine triphosphate (ATP) regulation, bone growth and digestion. Mn is the constituent of metalloenzymes such as manganese superoxide dismutase (Mn-SOD), arginase, pyruvate decarboxylase and glutamine synthetase (GS) [3].

Even though Mn is an element necessary for the proper functioning of cells and tissues in the body, its overabundance contributes to the pathophysiological processes of the central nervous system (CNS) [4]. Excessive Mn accumulation most prominently in the basal ganglia (globus pallidus, substantia nigra, subthalamic nuclei, caudate putamen) and in cortical structures, causes toxic effects and neurological brain disorder, referred to as manganism [5–7].

The biochemical mechanism responsible for Mn induced neurotoxicity (due to excessive Mn exposure) is still not fully elucidated. However, a potential mechanism for manganese neurotoxicity is manganese-induced oxidative stress, thereby leading to excessive production of reactive oxygen species (ROS). The ROS generate the oxidation of polyunsaturated fatty acids of the cell membrane, leading to its damage and consequently to cell death. Disturbing the pro-antioxidative balance in the organism is manifested by reduced activity of some antioxidant enzymes, inter alia, in the brain. Diminishing of ascorbic acid concentrations, whose presence within neurons and astrocytes in specific and efficient transporters, may also occur [8, 9]. Furthermore the manganese-induced ROS overproduction could affect the brain glucose metabolism—Mn can easily inhibit GS activity—one of the astrocytic-specific enzyme. The consequence of this process could be the enhanced biosynthesis of neurotransmitters such as glutamate (Glu), aspartate (Asp) and γ-amino butyric acid (GABA) [10, 11].

Glutamine (Gln) is the two-thirds amino acid in the brain. It plays an important role as a precursor of the neurotransmitter amino acids, including the excitatory Glu and Asp, and the inhibitory GABA and glycine (Gly). Glutamine is considered as an amino acid of conditional essentiality during certain diseases, where the demand for Gln outstrips its synthesis from endogenous precursors [12, 13]. Gln is required during catabolic processes to elicit optimal tissue responses to infection, inflammation and catabolism. Moreover, some researchers suggest that Gln metabolites can prevent against hydroxyl-radical apoptosis in animal erythrocytes [14]. Recently, Robinson et al. [15] showed that GS activity can be affected by hydrogen peroxide, providing the clear link between the occurrence of oxidative stress and glutamine synthesis.

We hypothesised that Gln supplementation could reduce manganese-induced oxidative stress and could influence the neurotransmission processes. The aim of this study was to investigate whether the long term administration of manganese (alone or in combination with glutamine) in dose and time dependent manner could affect the selected parameters of oxidative-antioxidative status as concentrations of malonic dialdehyde (MDA) and ascorbic acid (vitamin C) as well as the activity of antioxidant enzymes—superoxide dismutase (SOD) and glutathione peroxidase (GPx) in the brain of rats. Moreover, concentrations of excitatory (Glu, Asp) and inhibitory (GABA, Gly) amino acids were also determined.

## Materials and Methods

### Reagents

Kits for GPx Ransel and SOD (RANSOD) were obtained from Randox Laboratories Ltd., UK.

Reagents for amino acid analysis: ninhydrin, hydrindantin, 2-methoxyethanol, 4 M acetate buffer pH 5.6 and standards: physiological and glutamine were purchased from INGOS, Czech Republic. Citric acid monohydrate, lithium citrate tetrahydrate, lithium chloride and sodium azide were from Merck, Germany.

Manganese chloride and L-ascorbic acid were obtained Sigma Chemicals, USA. l-glutamine was obtained from POCH S.A. (Acros Organics), Poland.

All other reagents were of analytical grade and were provided by commercial suppliers.

### Experimental Design and Sampling Procedure

The experiments were carried out on 2-months-old albino male rats (Farm of Laboratory Animals, Warszawa, Poland) weighing 220–250 g at the beginning of the experiment. The animals were maintained under standard laboratory conditions (12 h light/dark cycle, room temperature 21 ± 1 °C) with free access to tap water as well as laboratory chow (Bacutil, Motycz, Poland), and were adapted to the laboratory conditions for at least 1 week. The study was carried out according to the National Institute of Health Guidelines for the Care and Use of Laboratory Animals and to the European Community Council Directive for the Care and Use of Laboratory Animals of 24 November 1986 (86/609/EEC), and was approved by the Local Ethics Committee on Animal Experimentation. All the animals were randomly divided into six groups (12 animals per each experimental group). The first five groups received deionised water, to which Mn or/and Gln was added; 1st group (*Mn300***)**—solution of manganese chloride at a concentration of 300 mg Mn/L, 2nd group (*Mn500***)**– solution of manganese chloride at a concentration of 500 mg Mn/L, 3rd group (*Gln***)**—solution of glutamine at a concentration of 4 g/L, 4th group (*Mn300*-*Gln***)**—solution of Mn at a concentration of 300 mg/L and 4 g Gln/L, and 5th (*Mn500*-*Gln***)**—solution of Mn at a concentration of 500 mg/L and 4 g Gln/L. The 6th group—control (*C*) received deionised water to drink in a daily manner. All the animals were fed with the same standard granulated rodent laboratory chow (Poland). Diet and fluids were offered ad libitum and their consumption was monitored daily over the experimental period.

The study period covered 6 weeks. Half of the animals (n = 6) of each group were intraperitoneally injected with 0.5 mL of 5 % ketamine after first 3 weeks. The remaining animals were euthanized after further 3 weeks. Before the euthanasia, all animals were fasting for 12 h, thus the last meal consumed by the experimental rats had no effect on results. After euthanasia the whole brain was removed and kept in −25 °C until amino acid analysis. The characteristic of study and control groups with statistical analysis is presented in Table 1.

**Table 1:** Body weight gain as well as intake of diet and water/fluids in the tested animals groups

Group	C	Gln	Mn500	Mn300	Mn500-Gln	Mn300-Gln
*Parameter*						
After 3 weeks						
Initial body weight (g)	222.0 ± 7.4	227.8 ± 8.1	235.8 ± 8.4	227.8 ± 14.3	235.6 ± 9.4	236.8 ± 14.0
Body weight after 3 weeks (g)	334.8 ± 16.4	325.8 ± 13.1	354.8 ± 15.4	325.3 ± 21.4	307.3 ± 13.6^^^	323.2 ± 17.2
Body weight gain (g)	112.8 ± 11.9	98.0 ± 10.6	119.0 ± 11.9	97.5 ± 17.9	71.6 ± 11.5*^,^^	86.4 ± 15.6
Food (g/rat/24 h)	28.4 ± 2.6	25.8 ± 3.4	29.8 ± 2.4	27.4 ± 3.4	24.8 ± 4.2	26.2 ± 2.2
Fluid (ml/rat/24 h)	42.8 ± 1.8^^^	36.7 ± 2.1*	43.0 ± 1.4	43.5 ± 1.2	37.4 ± 1.3^^,^*	39.4 ± 1.9
Mn (mg/rat/24 h)	–	–	24.1 ± 1.4	13.1 ± 1.7^^^^^	18.7 ± 2.2^^^	11.8 ± 1.8 ^^^^,~^
Gln (mg/rat/24 h)	–	146.8 ± 12.8	–	–	149.6 ± 14.8	157.6 ± 8.4
After 6 weeks						
Initial body weight (g)	218.7 ± 6.3	230.7 ± 9.6	240.0 ± 10.2	223.3 ± 11.3	234.0 ± 9.1	244.0 ± 17.1
Body weight after 6 weeks (g)	358.7 ± 10.7	358.6 ± 11.6	386.6 ± 12.8	335.6 ± 15.3^^^^	335.6 ± 11.4^^^^	355.8 ± 16.8
Body weight gain (g)	140.0 ± 8.5^^^	127.9 ± 10.6	146.6 ± 11.6	112.3 ± 13.4^^^	101.6 ± 10.2^^^,^*	111.8 ± 17.0
Food (g/rat/24 h)	27.4 ± 2.8	26.5 ± 2.2	29.9 ± 1.2	25.4 ± 3.1	23.6 ± 3.2	26.1 ± 3.8
Fluid (ml/rat/24 h)	44.6 ± 1.1	34.8 ± 1.8***	48.1 ± 2.8^###^	40.3 ± 1.2^#,^^^	38.2 ± 1.2**^,^^^^	38.9 ± 1.5*
Mn (mg/rat/24 h)	–	–	24.1 ± 1.2	12.1 ± 0.9^^^	19.1 ± 1.3^^^^	11.7 ± 0.7^~~~^
Gln (mg/rat/24 h)	–	139.2 ± 12.1	–	–	152.8 ± 11.6	155.6 ± 10.8

Data expressed as a mean ± SD

* significant different versus control group (* *p* < 0.05; ** *p* < 0.01; *** *p* < 0.001)

^#^ significant different versus Gln group (^#^ *p* < 0.05; ^###^ *p* < 0.001)

^ significant different versus Mn500 group (^^^ *p* < 0.05; ^^^^ *p* < 0.01; ^^^^^ *p* < 0.001)

^~^ significant different versus Mn500_Gln group (^~^ *p* < 0.05; ^~~~^ *p* < 0.001)

### Ion-Exchange Analysis of Amino Acid Concentrations in Rats’ Brain

To evaluate free amino acids concentration, six left and six right hemispheres from each group were homogenised and deproteinised in 6 % sulphosalicylic acid in lithium citrate buffer (pH 2.8) in 1:10 ratio. The homogenised samples were centrifuged 20 min at 12,000 rpm. The obtained supernatants were used for free amino acids determination with the use of ion-exchange chromatography [16] on an INGOS AAA 400 apparatus for automatic analysis of amino acids (Ingos Corp., Czech Republic). Amino acids were separated using analytic column OSTION LG FA (Ingos Corp., Czech Republic) and five lithium-citrate buffers (pH 2.6, 3.1, 3.35, 4.05 and 4.65, respectively). For amino acids evaluation was used the original software MIKRO version 1.8.0. (Ingos Corp., Czech Republic).

### Determination of Antioxidants in Rats’ Brain

Remaining hemispheres (six left and six right hemispheres) were homogenised in 0.1 mol/L Tris–HCl buffer (pH 7.4) in 1:10 ratio. The homogenised sample was centrifuged at 12000 rpm for 30 min at 4 °C. The supernatants were used for GPx, SOD, MDA and L-ascorbic acid determination.

GPx activity was determined by Paglia and Valentine method [17]. GPx catalyzes the process of glutathione (GSH) oxidation by cumene hydroperoxide. In the presence of glutathione reductase (GR) and NADPH the oxidized glutathione (GSSG) is immediately converted to the reduced form with a concomitant oxidation of NADPH to NADP^+^. GPx activity was expressed in units per gram of tissue (U/g of tissue).

SOD activity was determined according by Fridovitch and McCord method [18]. The method employs xantine and xantine oxidase (XOD) to generate superoxide radicals which react with nitroblue tetrazolium to form red formazan dye. The SOD activity is then measured by the degree of inhibition of the reaction. The enzyme activity in brain homogenates was expressed in units per gram of tissue(U/g of tissue) as a total SOD activity (the activity of all isoforms of this enzyme).

The ascorbic acid concentration in the rat brain was determined according to modified Kyaw’s method [19]. The basis of the method is the reduction of phosphotungstic acid by ascorbic acid from examined samples. The measurement of absorbance of the samples (blue tungstic oxide is formed—colloids of tungstic oxides) at 700 nm allows to calculate the concentration of ascorbic acid brain homogenates. The results were expressed in μmol/g of tissue.

The level of lipid peroxidation (LPO) was assayed in terms of MDA concentration which is the main product of this process. It was determined according to Ledwożyw’s method [20]. Briefly, 0.5 ml of tissue homogenate supernatant was mixed with 2.5 mL 1.22 M trichloroacetic acid (TCA) in 0.6 M HCl and allowed to stand for 15 min. Then 1.5 mL of 0.9 % thiobarbituric acid (TBA) was added and the mixture was incubated for 30 min in a boiling water bath. After cooling of 4 ml of n-butanol was added and the mixture was shaken variously. The samples were centrifuged at 1500 g for 10 min and then the absorbance of organic phase was measured with respect to blank (n-butanol alone). The concentrations of MDA in brain tissues were read from the standard curve obtained by using malonaldehyde bis-dimethylacetal and expressed in μmol/g of tissue.

The assays were performed using spectrophotometer HITACHI 2800 (Japan).

### Statistical Analysis

All statistical analyses were performed using STATISTICA program (version 10.0). The data were expressed as the medians of measured parameters. Shapiro–Wilk’s test was used to verify normality of data distribution and Brown–Forythe’s test to determine homogeneity of variances. Differences between groups were analyzed with Kruskal–Wallis one-way analysis of variances and analysis of variance (ANOVA) for multi-group comparison with post hoc Tukey’s test. For all analyses, *p* values <0.05 (*p* < 0.05) were considered as significant.

## Results

### Brain Tissue Glutamine Analysis

**Fig. 1:**
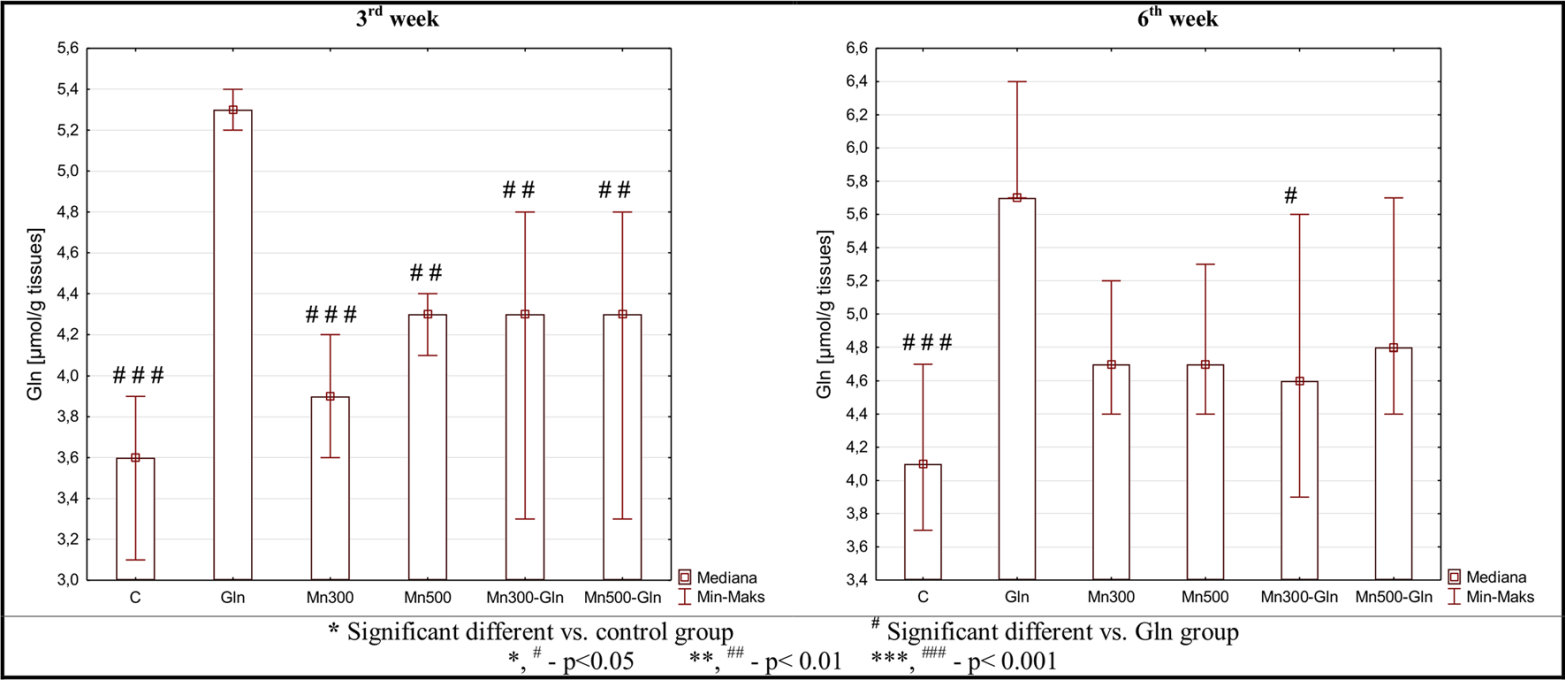
Concentration of glutamine in rats’ brains after 3 and 6 weeks of exposure to manganese and/or glutamine. Values presented as the median, minimum and maximum of examined parameters

After 3 weeks of experiment, glutamine concentration was statistically higher in the group of rats receiving only glutamine (Gln) in comparison to all other tested groups (C, Mn300, Mn500, Mn300-Gln, Mn500-Gln). Whereas, after 6 weeks of the experiment, glutamine levels were statistically higher in Gln group in comparison only to the control (C) and Mn300-Gln group (Fig. 1).

### Brain Tissues Oxidative-Antioxidative Status Parameters

The exposure of rats to Mn in drinking water contributes to diminishing of the antioxidant enzymes activity and the increase of the level of lipid peroxidation. GPx activity after 3 weeks of the experiment was significantly lower in rats receiving Mn at 500 mg/L dose in comparison to control group (C) and the group receiving only glutamine (Gln). However, after longer exposure time (6 weeks), the activity of this enzyme was diminished in both groups of rats receiving the manganese (Mn300 and Mn500) compared only to the control (C).

SOD activity in both stages of the experiment was decreased in the groups of rats receiving the manganese at 500 mg/L dose (either alone or in combination with glutamine—Mn500, Mn500-Gln) in comparison to the control and Gln groups.

The exposure of organisms to manganese appears to be the cause of increased lipid peroxidation, which is confirmed by an increased concentration of one of the indicators of this process –malondialdehyde. The level of this parameter was markedly higher in the group of rats receiving manganese at 500 mg/L dose in comparison to the control (C) after 3 and 6 weeks of the experiment and to the group of rats receiving only the glutamine (Gln) after 3 weeks. In addition, after 6 weeks, the concentration of MDA in Mn500 group was statistically higher than in the groups receiving both doses of manganese in combination with glutamine (Mn300-Gln and Mn500-Gln), which can indicate the protective influence of glutamine.

However, statistical analysis showed no distinct difference in vitamin C concentration between examined groups at both stages of the experiment.

**Fig. 2:**
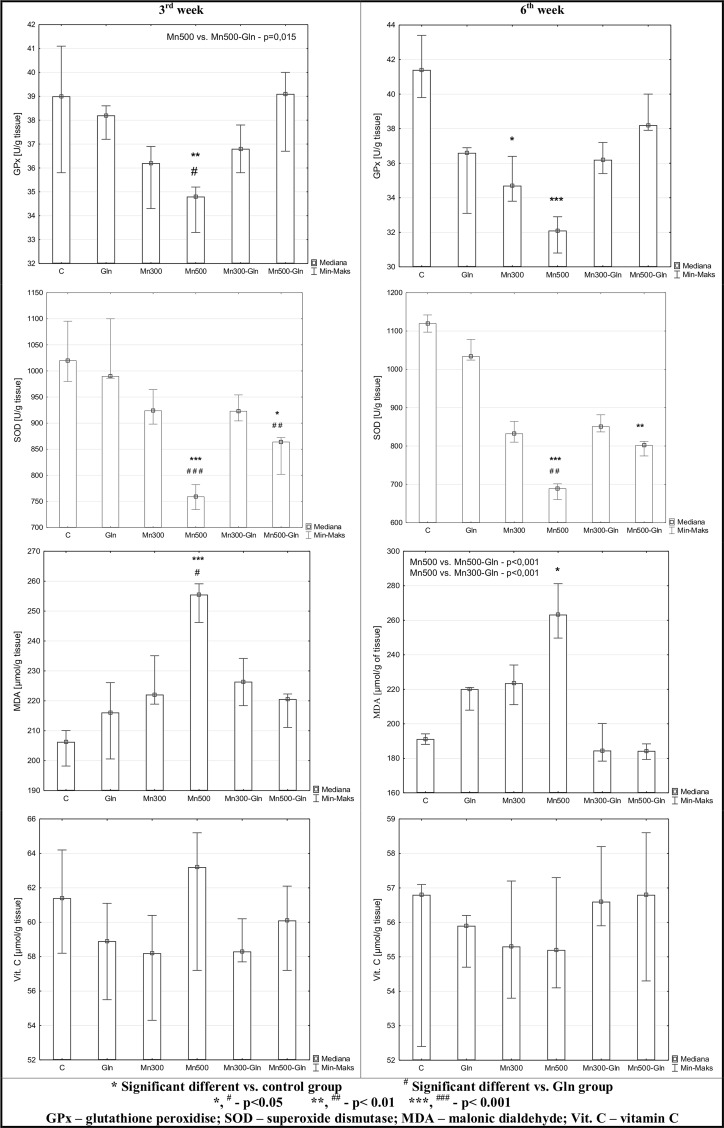
Effect of manganese and/or glutamine on chosen parameters of antioxidative-oxidative system in rats’ brain after 3 and 6 weeks of experiment. Values presented as a median, minimum and maximum of examined parameters

Effect of manganese and/or glutamine on oxidative-antioxidative status parameters in rats’ brain after 3 and 6 weeks of experiment was presented in Fig. 2.

### Brain Tissues Amino Acid Analysis

The most significant differences in examined amino acids levels in comparison to both control and Gln group were observed in the group of rats receiving Mn at 500 mg/L dose alone or with Gln.

Glu concentration was markedly increased in group receiving manganese at 500 mg/L after 6 weeks-long exposure. The obtained values were statistically different in comparison with both control and Gln-given groups.

Asp concentration was insignificantly increased in rats receiving manganese at 500 mg/L dose for 6 weeks versus groups of rats receiving manganese in combination with glutamine (Mn300-Gln and Mn-500-Gln).

GABA concentration values were significantly lower in Mn500 group compared to the Gln group, both after 3 and 6 weeks of experiment. Moreover, the level of this parameter was significantly decreased in groups of rats receiving Mn in both doses for 3 weeks versus groups receiving Mn plus Gln. After 6 weeks this difference was noticed only in the rats receiving manganese at higher dose.

Gly concentration was significantly increased in brain of rats receiving manganese at higher dose of Mn, both alone and in combination with Gln. The difference between these groups (Mn500 and Mn500-Gln) and groups: control and receiving Gln alone was observed after 3 weeks of experiment. After 6 weeks-long exposure only a slightly difference between groups receiving manganese at 500 mg/L dose (alone or with Gln) and the control group was noticed.

**Fig. 3:**
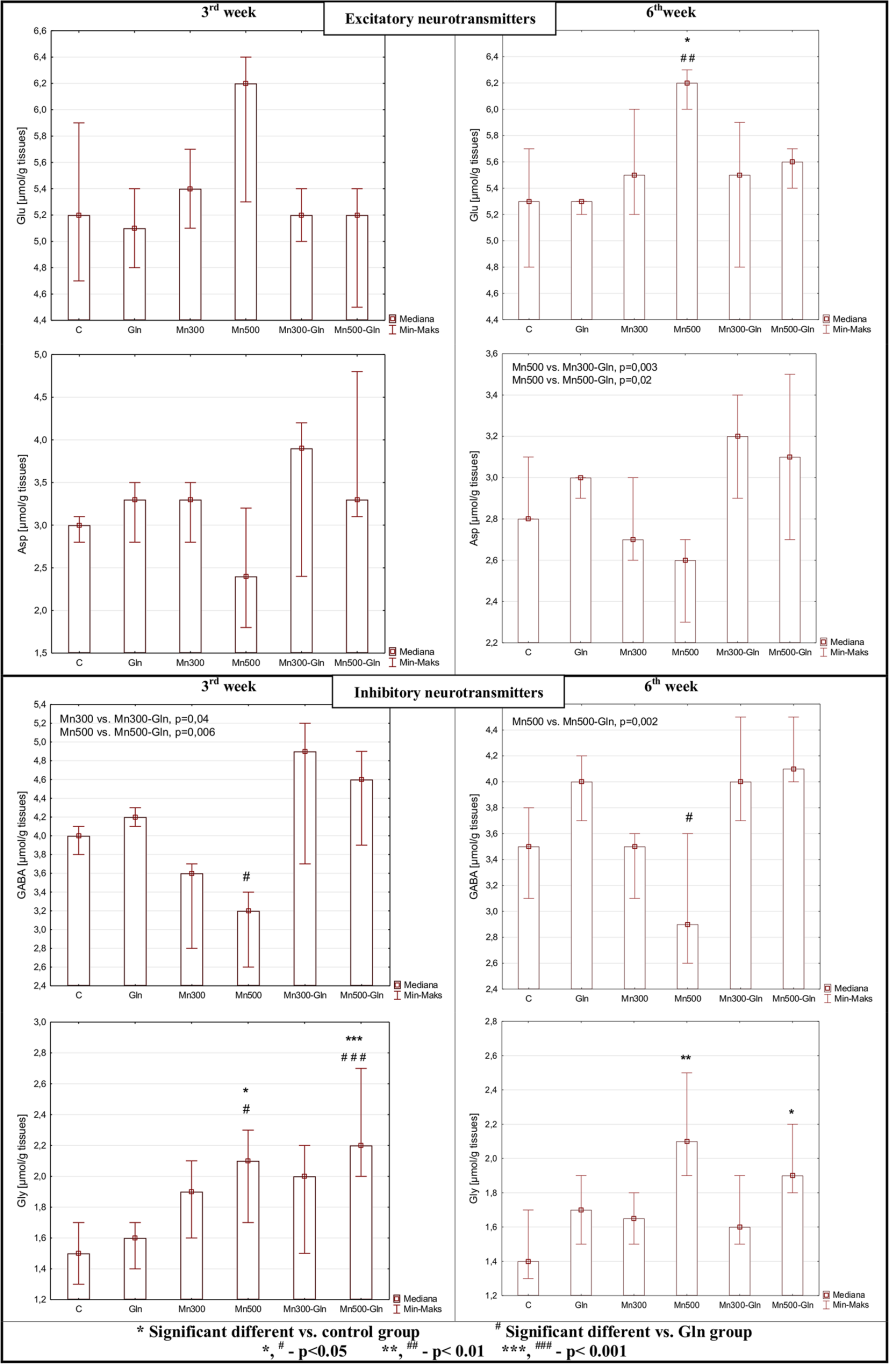
Effect of manganese and/or glutamine on exicatory (Glu, Asp) and inhibitory (GABA, Gly) neurotransmitter amino acids concentrations in rats’ brains after 3 and 6 weeks of experiment. Values presented as a median, minimum and maximum of examined parameters

The effect of manganese and/or glutamine on excitatory (Glu, Asp) and inhibitory (GABA, Gly) neurotransmitter amino acids concentrations in rats’ brain after 3 and 6 weeks of experiment is presented in Fig. 3.

## Discussion

### Brain Tissues Oxidative-Antioxidative Status Parameters

Manganese is an essential element for the proper course of many enzymatic reactions. However, depending on the concentration, Mn can exert protective or toxic effect. Moreover, it could be a scavenger of superoxides or induce their production, which in consequence may disrupt the antioxidant system. Excessively high systemic exposure to manganese has been recognized as neurotoxic. Pathophysiology of Mn-associated neurotoxicity was observed in mammals in both the acute and chronic exposures [21, 22]. Available data demonstrated that Mn exposure stimulated generation of ROS and induced a decrease in the activity of SOD and GPx [2, 23–29], but the mechanism of this process is still not fully elucidated.

In the present study, statistically lower SOD activity in the group of rats receiving higher Mn dose, regardless the duration of the experiment, was observed. Glutamine in the diet admittedly increases SOD activity, but it is unable to restore the intracellular redox balance. On the other hand, GPx activity also depends on the dose and the duration of Mn exposure. After 3 weeks, a higher dose of Mn diminished the GPx activity, while longer exposure contributes to the reduction of this enzyme activity even at lower doses of the metal. Glutamine administered in the diet contributes to establishing the status of the balance, regardless of the time of exposure and the applied dose of Mn.

In the available studies there is no information about the interaction of glutamine and manganese administered in the diet and the mechanism according to which glutamine could protect against Mn-induced toxicity. However, Roth et al. [30] showed that Gln is the precursor for the synthesis of GSH and presented significant correlation between Gln supplementation and intercellular GSH content. Therefore, we suppose that glutamine may increase the GPx activity in the brain of rats exposed to Mn by providing an energy source and by acting as precursor of glutathione. Furthermore, our findings are consistent with the results of other researchers according to which the Gln supplementation enhances antioxidant capacity in various diseases [2, 31–35].

Noteworthy is that excessive O_2_^**−**^ and H_2_O_2_ production, and consequently increased HO^·^ expression, may result in the lipid peroxidation (LPO). In vitro studies [7, 36] indicated that the increase in LPO and depletion of high energy phosphates are the first factors indicating the astrocyte and neuronal dysfunction caused by Mn. In our research LPO was determined by measuring MDA concentration. We noticed that the peroxidation process occurs in groups of rats exposed to 500 mg Mn/L after 3 weeks of exposure. Interestingly, the effect of glutamine co-administration is observable after 6 weeks of experiment—lipid peroxidation in groups of rats receiving Mn + Gln is significantly lower than in the groups of animals receiving metal alone. Previous observation revealed that the administration of glutamine completely prevented the increase in thiobarbituric acid reactive substances (TBARS) content in rats’ liver treated with cobalt [31, 37].

Ascorbic acid, a non-enzymatic antioxidant, is found in high concentrations in the brain and is an essential vitamin for its physiological functions [38]. Zhang et al. [25] studies showed that the ascorbic acid prevents the production of ROS in mitochondrial preparations caused by high doses of manganese. According to these authors, ascorbic acid donates hydrogen atom to the hydroxyl radicals, singlet oxygen and peroxide and consequently causes blocking of free radical chain reactions, particularly in neurodegenerative diseases [39, 40]. However, in vitro studies demonstrated vitamin C prooxidative properties—ascorbic acid can generate the formation of free radicals by Fenton reaction [41, 42]. Similar conclusions from an in vivo study were put forward by Aydogan et al. [43]. These authors suggest that ascorbic acid can exert prooxidant action (especially in the brain, which is rich in Fe^2+^ ions) leading to neurotoxicity. In our study no statistically significant changes in the concentration of ascorbic acid, irrespective of the dose and time of manganese intoxication, were stated. Therefore determination the influence of manganese on the level of ascorbic acid in the brain is quite difficult. However, during examination of the ascorbic acid concentration in the brain, another mechanism of its action (not only associated with its antioxidant activity) should be considered. Ascorbic acid acts as antagonist in the glutamatergic and dopaminergic systems. It is released from glutamatergic neurons in the glutamate reuptake process, in which the glutamate transporter exchanges ascorbic acids for glutamate [44]. The balance between glutamate and ascorbic acids can regulate neuronal capability to degeneration. The increase of the glutamergic activity can result in the accumulation of reactive oxygen species [39].

### Brain Tissues Amino Acids

Manganese has been recently showed to exert effect on neurotransmitter amino acids concentrations in the brain. Obtained results depend on variety of different factors such as the mode and time of exposure and the amount of the applied dose of the metal. In vitro and in vivo studies indicate that there is a relationship between excessive accumulation of manganese versus fluctuation of GABA concentration, although the results of these studies are inconclusive [45–47]. Lipe et al. [45] noticed the increased level of GABA, as well as of other neurotransmitters: aspartate, glutamate and glutamine in the cerebellum of the adults rats (90-day-old) exposed to 20 mg Mn/kg per day for 30 days. Moreover, in this study, significant decrease in the concentration of glutamine in caudate nucleus and hippocampus of weanling (30-day-old) rats dosed with 10 mg/kg per day for 30 days was observed. However, Bonilla et al. [48] observed no alterations in concentration of GABA and other inhibitory and excitatory amino acids in the striatum of mice treated with intraperitoneal Mn injections (5 mg/kg body weight per day) during nine weeks while reducing the contents of aspartate, glutamate, GABA and glicyne in olfactory bulb. Santos et al. [46] showed no effect of sub-chronic exposure to manganese injection on GABA, Glu and Asp concentrations and increase of Gly concentration in whole rats’ brain in this condition. On the other hand, Takeda et al. [47] showed that manganese reduces the level of glycine in the striatum of rats injected with Mn. Santos et al. [46] explain their findings that the concentration of these amino acids determined in the entire brain masks changes in those areas of the brain where amino acids accumulation is the greatest (globus pallidus and the striatum). However, our research has shown that the manganese toxicity causes changes in the concentrations of neurotransmitters in whole rats’ brain. We noticed that higher dose of Mn after 3 weeks of exposure caused a slight increase in the concentration of Gly, Gln and Glu and reduction in the concentration of GABA in comparison to the control. Administration of Mn in 500 mg/L dose (alone or in combination with Gln) resulted in an increase in Gly concentration. We presume that Gly, as a precursor of GSH, can protect against Mn-induced oxidative stress.

There are several explanations for the relationship between manganese neurotoxicity and neurotransmitter amino acids concentration in the brain. Glu and GABA are the most abundant neurotransmitters in the brain and their metabolism is closely related [49, 50]. Mn neurotoxicity may be related to an indirect excitotoxic event caused by the increased extracellular Glu levels. Bouabid et al. [49] elucidates that Mn is concurrently released with Glu from glutamatergic neuron terminals. Therefore, the hyperactivity of corticostriatal neurons during Mn intoxication may partially contribute to the elevated extracellular Glu levels. Moreover, Mn may be stimulator of post-synaptic receptors to Glu. In addition, this element excitotoxicity can result in a reduced ability of astrocytes to clear Glu from the synapse and Glu/aspartate transporter expression in these cells what can lead to increased Glu concentration [49, 50]. Furthermore, several studies have demonstrated the tendency of Mn to impair components of the glutamine/glutamate-GABA cycle what leads to reduction in Glu uptake and elevation in extracellular Glu level [11]. Gln is formed in Glu and ammonia reaction catalyzed by GS. The newly formed glutamine is transferred from astrocytes to neighbouring neurons and hydrolized by phosphate-activated glutaminase (PAG) which results in a Glu formation. Part of this Glu could undergo decarboxylation to GABA (using glutamate decarboxylase, GAD), transamination to Asp or it could be converted to the tricarboxylic acid (TCA) cycle intermediate—α-ketoglutarate [12]. In turn, Glu released from neurons can be transported to the astrocyte via glutamate transporter, where it is amminated to Gln. This creates a shuttling metabolic cycle defined as glutamine/glutamate-GABA cycle (GGC) [51]. Several studies have demonstrated the tendency of Mn to impairing components of the GGC leading to reduction in Glu uptake and elevation in extracellular Glu level [52].Thus, manganese toxicity results in inhibition of GS activity, which can cause decrease in neuronal energy level and synthesis of glutamine diminish. Moreover, in the animal models, ROS can inhibit GS resulting in the decrease of Gln synthesis [15]. Finally, the result is leading to disturbances of Gln/Glu-GABA cycle, elevation of Glu and the decrease of Gln concentration. Although, in our study statistically significant diminish concentration of Gln in Mn-treated groups in comparison to the control was not observed.

Earlier studies have shown that dietary glutamine can be taken into the brain, via the Na^+^-dependent N-transportation system, leading to the increase of Gln level in the brain [52, 53]. This appears to have been confirmed in our research which showed that exogenous glutamine can elevate glutamine level in the rats’ brain. The observed trend toward time-dependent GABA increase in brains of Gln group suggests that exogenous glutamine does not disrupt the Gln/Glu-GABA cycle, but after entrance to neurons, it is directly deaminated by phosphate-activated glutaminase (PAG) to glutamate, and then Glu is converted by GAD to GABA.

### Summary

In summary, the presented studies established that Mn, depending on the dose and duration of exposure, generates excessive production of ROS, which manifests itself in the oxidative stress intensification. Consistently with increased ROS production, we observed decrease in GPx and SOD activity and increase in MDA level. Gln is effective in reducing Mn-induced oxidative stress. The consequence of reduction in ROS production is an increase in GPx activity and reduction in MDA concentration observed in in Mn-Gln co-treated groups of rats. Moreover, the presented experiment indicates that manganese influences the concentration of neurotransmitters’ amino acids.
